# Metabolomic effects of intrauterine meloxicam perfusion on histotroph in dairy heifers during diestrus

**DOI:** 10.3389/fvets.2025.1528530

**Published:** 2025-03-18

**Authors:** Beibei Zhang, Yuan Han, Ming Cheng, Longgang Yan, Kangkang Gao, Dong Zhou, Aihua Wang, Pengfei Lin, Yaping Jin

**Affiliations:** ^1^Key Laboratory of Animal Biotechnology of the Ministry of Agriculture, College of Veterinary Medicine, Northwest A&F University, Yangling, Shaanxi, China; ^2^Department of Clinical Veterinary Medicine, College of Veterinary Medicine, Northwest A&F University, Yangling, Shaanxi, China; ^3^Department of Preventive Veterinary Medicine, College of Veterinary Medicine, Northwest A&F University, Yangling, Shaanxi, China

**Keywords:** histotroph, meloxicam, prostaglandins, untargeted metabolomics, conceptus elongation

## Abstract

In ruminants, conceptus elongation is a crucial developmental process that depends on uterine lumen fluid (ULF) and coincides with a period of high pregnancy loss. Prostaglandins (PGs) play indispensable roles in conceptus elongation and implantation. However, the effects of uterus-derived PGs on the uterine environment remain unclear. To explore the metabolic pathways and metabolites induced by endometrium-derived PGs that may affect conceptus elongation and implantation in dairy cows, we investigated the biochemical composition of ULF following intrauterine perfusion of meloxicam from days 12 to 14 of the estrous cycle. Intrauterine administration of meloxicam significantly downregulated the prostaglandin-related metabolites in the ULF. A total of 385 distinct metabolites, primarily clustered within lipids and lipid-like molecules, organic acids and derivatives, organoheterocyclic compounds, and benzenoids, were identified. The metabolite network analysis identified 10 core metabolites as follows: S-adenosylhomocysteine, guanosine, inosine, thymidine, cholic acid, xanthine, niacinamide, prostaglandin I2, 5-hydroxyindoleacetic acid, and indoleacetaldehyde. The pathway enrichment analysis revealed three significantly altered metabolic pathways: arachidonic acid metabolism, tryptophan (Trp) metabolism, and linoleic acid metabolism. A total of five metabolites—guanosine, inosine, thymidine, butyryl-l-carnitine, and l-carnitine—were associated with attachment and pregnancy loss and could serve as predictors of fertility. This global metabolic study of ULF enhances our understanding of histotroph alternations induced by uterus-derived PGs during diestrus in dairy cows, with implications for improving dairy cow fertility.

## 1 Introduction

Fertility is crucial for maintaining regular calving cycles and efficient milk production (1). Subfertility is a common issue in dairy cows (2). A key factor affecting fertility is embryo loss during the first month of pregnancy due to the inability of the uterus to support embryo growth and implantation (3, 4, 62). In cattle, spherical blastocysts in bovine hatch from the zona pellucida on days 9–10 after insemination (day 0), subsequently developing into ovoid or tubular forms on days 12–14. These are defined as conceptuses, which include the embryo-fetus and associated extraembryonic membranes (5–7). The conceptus undergoes rapid growth and elongates into a filamentous form during the elongation period, which can reach more than 25 cm and occupy the entire length of the uterine horn (3, 8).

The development of the preimplantation conceptus is fundamental to a successful pregnancy, and the failure of this process is strongly associated with reduced fertility in dairy cows (3). The length of the conceptus has been linked to the ability of the uterus to support conceptus development and implantation (3). Conceptus development in ruminants cannot occur in the absence of uterine glands (7, 9) or *in vitro*, as it is highly dependent on uterine lumen fluid (ULF) (5, 10–12). Therefore, a comprehensive understanding of environmental factors that support embryo development and successful implantation is key to improving fertility in dairy cows.

ULF contains a variety of substances to support conceptus survival, growth, and implantation, collectively termed histotroph (11, 12). It is primarily derived from nutrients secreted/transported into the uterine lumen by the endometrial luminal epithelium (LE) and glandular epithelia (GE), influenced by progesterone (P_4_) and some secreted signaling factors, such as interferon tau (IFNT) and prostaglandins (PGs), from the peri-implantation conceptus and/or endometrium (5, 6, 13, 70). In ruminants, IFNT is secreted by the conceptus and acts on the endometrium to inhibit the release of luteolytic pulses of PGF_2α_, thereby ensuring the maintenance of the corpus luteum (CL) and circulating P_4_ concentrations (14, 15). PGs are important mediators of endometrial responsiveness to P_4_ and IFNT during early pregnancy, regulating gene expression associated with elongation and implantation in the endometrial epithelium prior to pregnancy recognition (16, 17). However, alternations in the PGs-regulated uterine environment during early pregnancy in dairy cattle remain unclear.

In ruminants, PGs regulate multiple reproductive processes, especially conceptus development and implantation, which are important mediators of maternal endometrial responses to pregnancy signaling molecules (5, 18). The content of PGs in ULF rapidly increases from days 12 to 18 of pregnancy and the estrous cycle in dairy heifers, corresponding to the period of embryo development (19). The level of PGs in ULF and the expression of prostaglandin-related genes in the endometrium are linked to fertility variations (20, 21). Compared to high fertility heifers, PGs content in ULF and the expression of prostaglandin-endoperoxide synthase 2 (PTGS2) mRNA in the endometrium are much lower in subfertile heifers (22). Cyclooxygenases (COXs) mediate the conversion of arachidonic acid to PGs (23). COX-2, the dominant isoform, is expressed in the endometrium during early pregnancy and the estrous cycle, with its expression regulated by P_4_ and IFNT in cattle (24). Intrauterine infusions of meloxicam (a selective inhibitor of COX-2 that is 13.1 times more effective at inhibiting COX-2 compared to COX-1) prevent conceptus elongation in sheep (17, 25) and reduce pregnancy rates in heifers when administered on day 15 of gestation (26). However, there is no effect on conceptus development when COX-2 is downregulated in embryos using CRISPR-Cas9 genome editing (27). These findings suggest that intrauterine infusions of meloxicam during conceptus elongation may inhibit the elongation by altering ULF, possibly through COX-2 downregulation in the endometrium. Therefore, it is necessary to study the effects of uterus-derived PGs on the uterine environment, which can help us understand the mechanisms of conceptus development and provide potential strategies to improve fertility.

This study investigated the effect of uterus-derived PGs on histotroph on days 12–15 of the estrous cycle in dairy heifers. Untargeted metabolomics analysis was employed to identify molecules potentially improving conceptus development and to gain new insights into the mechanisms by which PGs regulate the uterine environment. We hypothesized that the intrauterine infusion of meloxicam reduces the abundance of PGs in uterine fluid, which further causes changes in the content of metabolites in the ULF related to pregnancy and conceptus development.

## 2 Materials and methods

### 2.1 Animals

All experiments in this study were carried out in accordance with the Guide for the Care and Use of Agricultural Animals in Agricultural Research and Teaching and were approved by the Ethics Committee on the Use and Care of Animals at Northwest A&F University (Ethical Approval number: No. 2021100903).

### 2.2 Study design

Holstein heifers [12 ± 2 months; body weight (BW): 360 ± 30 kg; body condition score (BCS): 3.0 ± 0.25] were housed in a pasture and fed a young cow total mixed ration (TMR) once daily.

**Figure 1:**
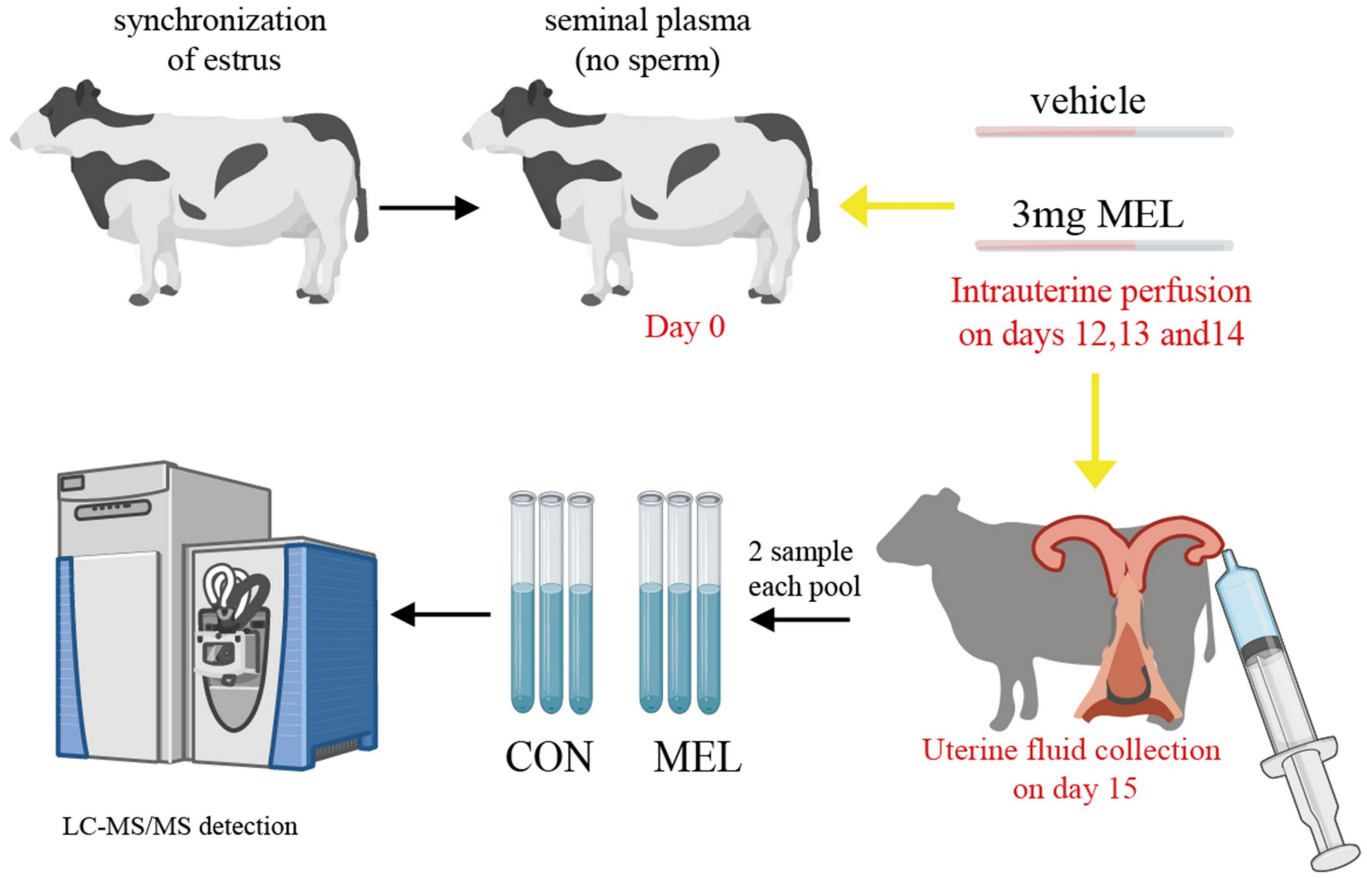
Schematic diagram of the treatment and collection of uterine fluid. The dairy heifers had their estrous cycle and ovulation synchronized, and thirteen heifers were assigned randomly to two groups. The CON group (*n* = 6) was intrauterine perfused with the same volume of PBS, and the MEL group (*n* = 6) received 3 mg of meloxicam on days 12, 13, and 14 of the estrous cycle (the window of conceptus elongation). Uterine lumen fluid samples were collected on day 15 and divided into three pools in each group, with two samples in each pool. Then, the uterine lumen fluid samples were characterized using LC–MS/MS. CON, vehicle; MEL, meloxicam; LC–MS/MS, liquid chromatography tandem mass spectrometry; PBS, phosphate buffer saline.

All heifers were subjected to an estrous cycle synchronization program, as previously described by Simintiras et al. (28). In brief, the day of the last injection in the synchronization program was considered study day 0. On this day, the heifers were inseminated with sperm-free seminal plasma, which was obtained by removing sperm through centrifugation at 4000 rpm. Transrectal ultrasonography was used to examine the ovaries with B-ultrasound (7.5 MHz Line Array Probe, IMV Technologies Group). The heifers that had a dominant follicle and no CL on day 0 and subsequently had a CL on the same ovary on day 7 were utilized. The heifers with successful synchronization of the estrous cycle (*n* = 12) were randomly allocated to one of two groups for intrauterine perfusion, as described in Figure 1. At the same time, on days 12, 13, and 14 of the estrous cycle, twelve heifers underwent uterine infusion of either meloxicam (*n* = 6) or a vehicle (*n* = 6), with the infusion placed in the lumen of the uterine horn ipsilateral to the corpus luteum. Meloxicam (Sigma, USA) was dissolved in 300 uL DMSO and then diluted with 5 mL PBS, while the vehicle had the same preparation without meloxicam. The dose of MEL was determined according to a previous report (17).

### 2.3 Uterine lumen fluid collection

On day 15 of the estrous cycle, the heifer was injected with a 2% lidocaine HCL solution (Sichuan Jixing Animal Pharmaceutical CO., LTD, China) in the first coccygeal intervertebral space. Then, uterine fluid was collected from the uterine horn ipsilateral to the CL, according to a previous report (29) with some modifications. The uterine horn was flushed via transcervical catheterization using an embryo transfer gun connected to a 50-mL syringe containing 30 mL PBS. Then, the uterine lumen fluid was recovered into a 100-mL syringe and immediately transferred to a sterile tube. The samples with a recovered volume exceeding 15 mL and free from visible blood contamination were centrifuged at 4°C, 2000 × *g* for 20 min.

### 2.4 Metabolome extraction

For metabolic analysis, 50 μL of the ULF samples were thawed on ice and mixed with a 4-fold extraction buffer, MeOH/ACN (1:1, v/v). After fully vortexed and sonicated, the samples were precipitated at −20°C for 1 h, then centrifugated at 4°C at 18000 × *g* for 15 min. The supernatants were transferred to a new centrifuge tube and drained using a concentrator. Afterward, the dried extracts were redissolved in an equal volume of CAN:H_2_O (1:1, v/v) using an ultrasonic device and again centrifuged at 4°C, 18000 × *g* for 15 min. The supernatants were transferred to a new centrifuge tube for liquid chromatography triple quadrupole mass spectrometry (LC–MS) analysis.

### 2.5 Untargeted metabolomics using LC–MS/MS

The sample extracts were separated using the Waters ACQUITY UPLC ultra-high-performance liquid phase system, injected into the capillary ion source for ionization, and analyzed using the timesTOF Pro mass spectrometry system. The ion source voltage was set to +5.5 kV in the positive ion mode and − 4.50 kV in the negative ion mode, and the parent ion of the peptide segment and its secondary fragments were detected and analyzed using high-resolution TOF. The secondary mass spectrometry scan range was set from 50 to 1,300. The data acquisition mode used the parallel cumulative serial fragmentation (PASEF) mode. A secondary spectrum with a charge number of parent ions in the range of 0–1 was collected using the PASEF mode twice after the primary mass spectrometry collection. The dynamic exclusion time for the series of mass spectrometry scans was set to 6 s to avoid repeated scanning of the parent ions.

### 2.6 Data preprocessing and annotation

MetaboScape 2022 was used for the peak extraction, peak alignment, and retention time correction of the raw data, and the primary and secondary mass errors were controlled within 20 ppm to ensure the accuracy of the identification results. The final identification of these compounds was performed by matching their fragmentation spectra with reference spectra from curated databases, using Progenesis QI software, the online METLIN database,^1^ and Biomark’s self-built library. Missing values were widely present in the LC–MS-based metabolomics data and affected the normality and variance of the data. In this study, the k-nearest neighbor method (30) was used to manage the missing values.

### 2.7 Data analysis

The intragroup aggregation and intergroup separation tendencies were determined using principal component analysis (PCA), and the intergroup differences were further examined using orthogonal partial least squares discriminant analysis (OPLS-DA). Differentially altered metabolites were screened in CON versus MEL based on variable importance in projection (VIP ≥ 1), a *p*-value <0.05, and a fold change (FC) value ≥1.5 or FC ≤ 0.667. Heatmap visualization was performed using the R package pheatmap. Metaboanalyst 6.0^2^ was used to identify and visualize the enriched metabolic pathways (31).

### 2.8 Statistical analyses

Statistical analyses of the changes in the corpus luteum and follicles in the ovaries between the two groups were performed using GraphPad Prism (version 8.0) with unpaired Student’s *t*-test for experiments. The results with statistically significant differences are indicated by asterisks (*p* < 0.05 denoted by *, *p* < 0.01 denoted by **, and *p* > 0.05 denoted by ns).

## 3 Results

### 3.1 Ultrasonographic assessment of the corpus luteum and follicle in the ovary

**Figure 2:**
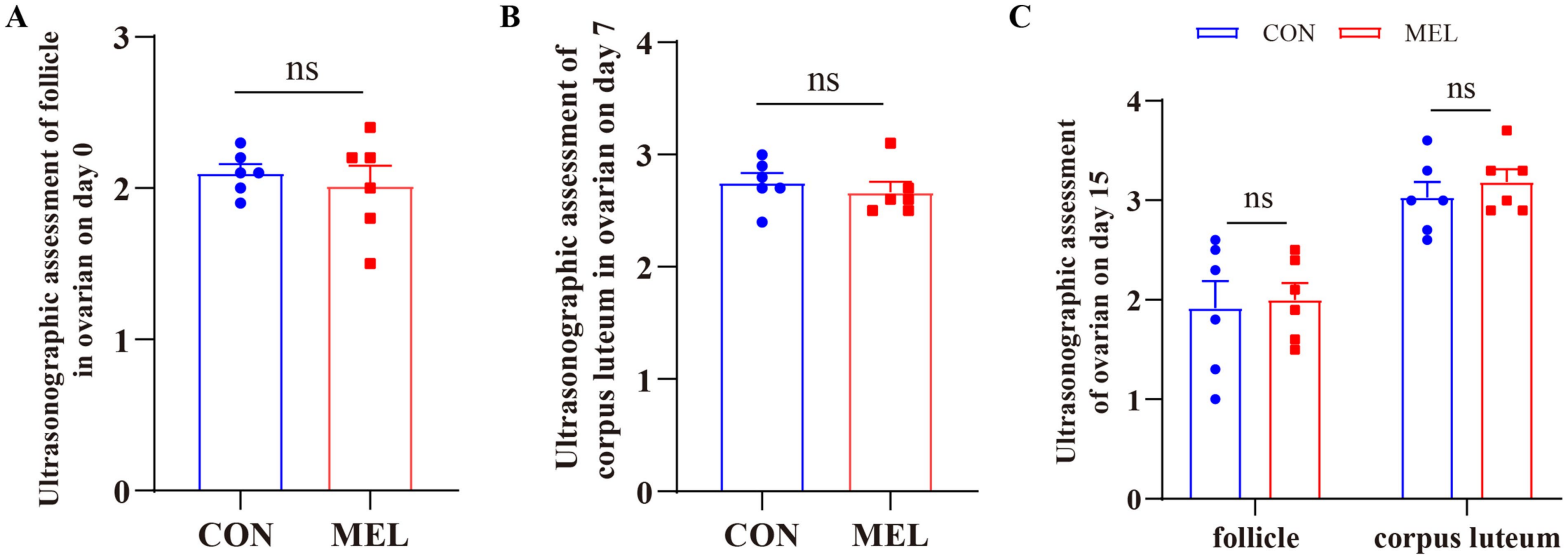
Changes in the ovarian structures in the heifers at different time points during the estrous cycle. **(A)** Transrectal ultrasonography assessment of the follicle in the ovary on day 0 of the estrous cycle using B-ultrasound. **(B)** Changes in the corpus luteum (CL) in the ovary on day 7 of the estrous cycle. **(C)** Changes in the follicle and CL in the ovary on day 15 of the estrous cycle.

Before the intrauterine perfusion and the collection of the uterine fluid, the corpus luteum (CL) and ovarian follicles were evaluated using transrectal ultrasonography (US) on days 0, 7, and 15 of the estrous cycle. The results are shown in Figure 2 and Supplementary Table S1. The transrectal ultrasonography showed that the bilateral ovaries of all dairy heifers did not contain any CL but displayed a dominant follicle of approximately 1.5–2.4 cm on day 0, and there was no significant difference between the CON and MEL groups (*p* = 0.5775) (Figure 2A). On day 7, the ovaries contained corpora lutea, which were classified as either homogenous (CL_hom_) or cavity-containing (CL_cav_), ranging in size from 2.4 to 3.3 cm, along with several follicles <0.5 cm. Compared to the CON group, there was no significant difference in the CL size on day 7 (*p* = 0.5199) (Figure 2B). On day 15 of the ULF collection, the US examination showed that the ovaries contained corpora lutea ranging from 2.5 to 3.7 cm and follicles ranging from 1.3 to 2.6 cm. The intrauterine meloxicam infusion did not significantly affect the ovarian CL (*p* = 0.9406) and follicle (*p* = 0.8215) size (Figure 2C).

### 3.2 Intrauterine perfusion of meloxicam altered the metabolite profiles of the ULF

**Figure 3:**
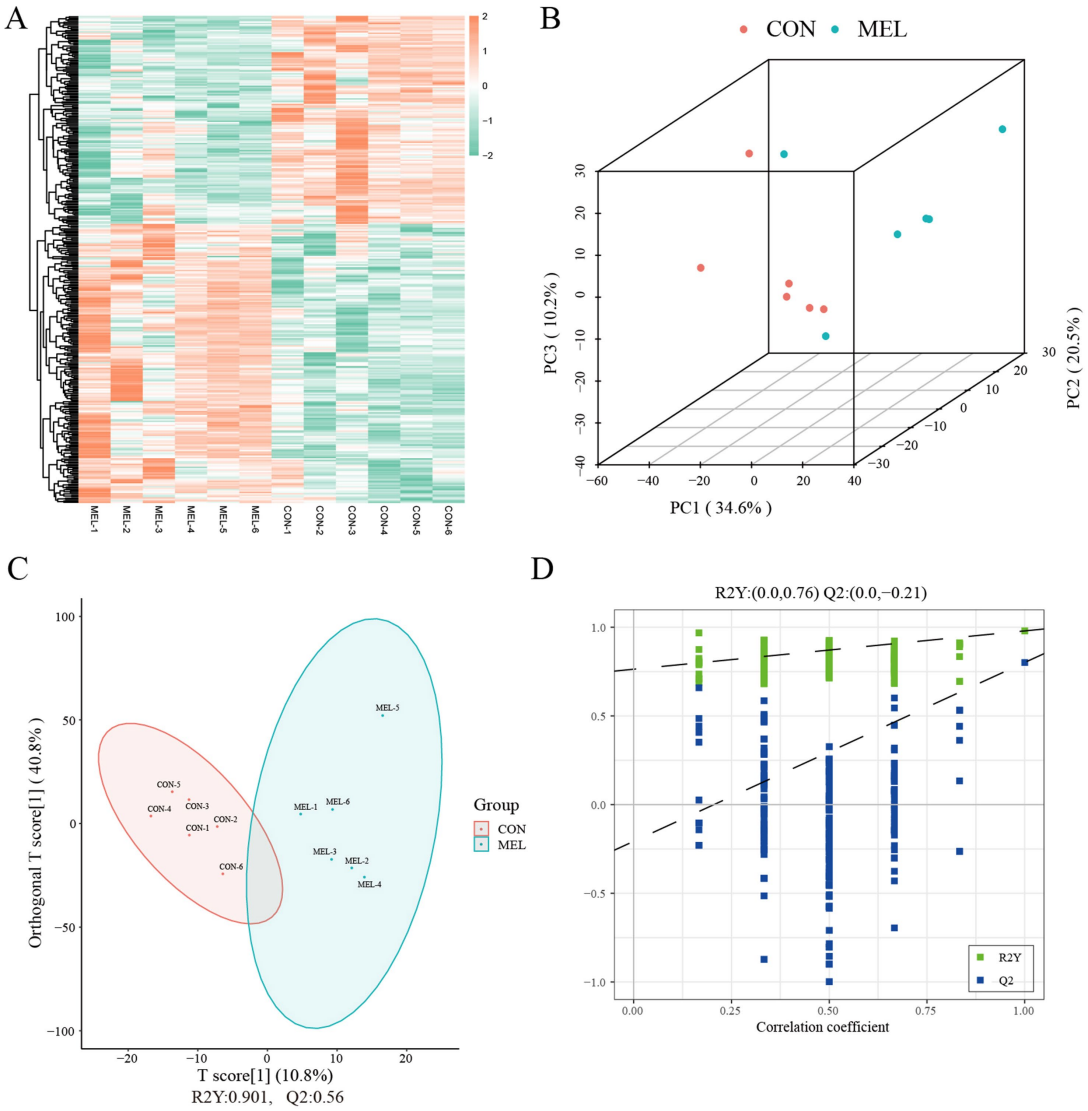
Metabolite profiles of the uterine lumen fluid in the dairy cows. **(A)** Heatmap showing the hierarchical clustering and molecular features of the ULF from the dairy cows treated with meloxicam. **(B)** Scatter plot showing the principal component analysis (PCA) of the ULF metabolomics. **(C)** Score plot of the orthogonal partial least squares discriminant analysis (OPLS-DA). **(D)** Permutation test plots between the CON and MEL groups. Q2 = percentage of Y dispersions predicted by the model using cross-validation; R2 = percentage of Y dispersions explained by the model. ULF, uterine lumen fluid; CON, vehicle; MEL, meloxicam.

A total of 1,419 metabolites were identified in the ULF in the positive ion mode, and 557 metabolites were detected in the negative ion mode (Supplementary Table S2). The untargeted metabolomic analysis of the ULF revealed distinct molecular features of the metabolites (Figure 3A). The PCA score plot showed excellent separation between the CON and MEL groups (Figure 3B). We further applied OPLS-DA to examine the specific characteristics of the metabolites between the CON and MEL groups. The score plot consistently revealed the distribution of each sample between the CON and MEL groups (Figure 3C), which were distinguished by R2Y = 0.946 and Q2 = 0.727. The intercepts (R2, Q2) from permutation testing further confirmed the reliability and stability of the OPLS-DA model (Figure 3D). The differences between the CON and MEL groups were identified using multivariate analyses based on the untargeted metabolomics.

### 3.3 Intrauterine infusion of MEL downregulated the abundance of the prostaglandin-related metabolites in the ULF

**Figure 4:**
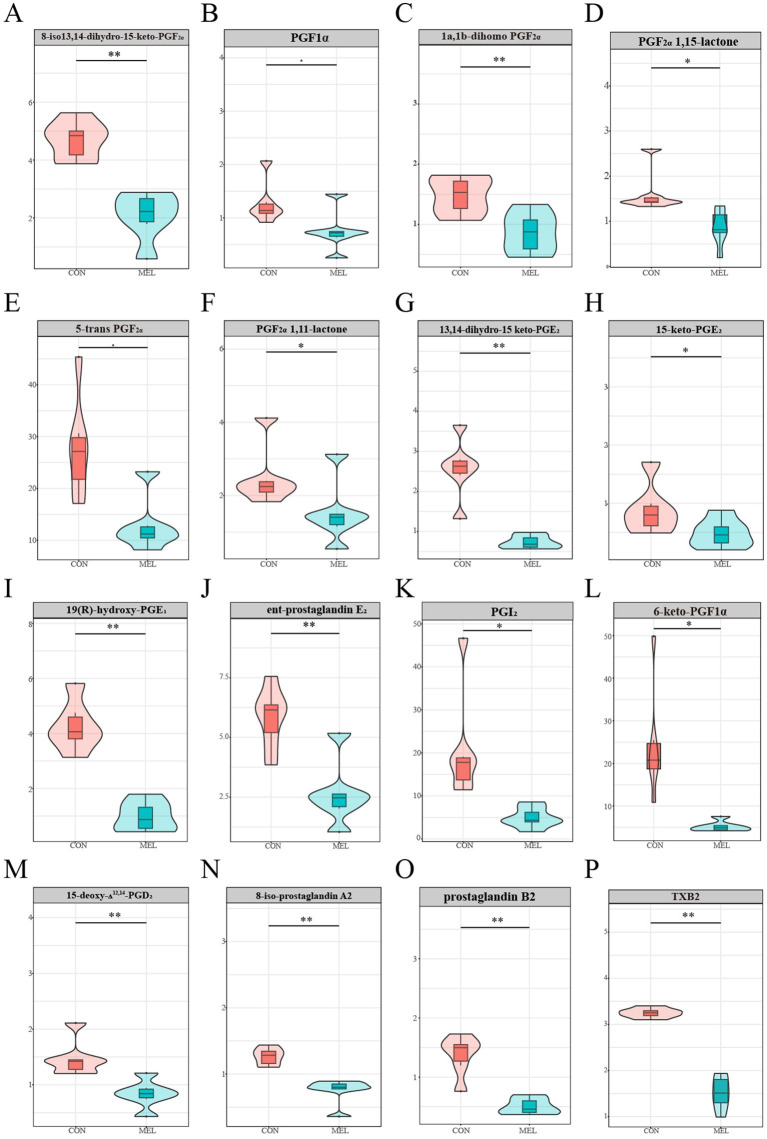
Prostaglandins-related metabolites in the uterine lumen fluid. The relative abundance between the CON and MEL groups **(A)** 8-iso-13,14-dihydro-15-keto-PGF2α, **(B)** prostaglandin F1α, **(C)** 1a,1b-dihomo PGF2α, **(D)** PGF2α 1,15-lactone, **(E)** 5-trans PGF2α, **(F)** PGF2α 1,11-lactone, **(G)** 13,14-dihydro-15-keto-PGE_2_, **(H)** 15-keto-PGE_2_, **(I)** 19(r)-hydroxy-PGE1, **(J)** *ent*-prostaglandin E_2_, **(K)** PGI2, **(L)** 6-keto-PGF1α, **(M)** 15-deoxy-Δ12,14-PGD2, **(N)** 8-iso-prostaglandin A2, **(O)** prostaglandin B2, **(P)** TXB2.

To further demonstrate that intrauterine infusion of meloxicam inhibits the synthesis of PGs in the uterus, we analyzed the most relevant PGs and prostaglandin-related metabolites in the ULF. As shown in Figure 4, the content of the prostaglandins and related metabolites in the ULF was significantly reduced following intrauterine meloxicam infusion. The main metabolites of PGF_2α_, including 8-iso-13,14-dihydro-15-keto-PGF_2α_ (*p* = 0.0041), prostaglandin F1α (*p* = 0.0393), 1a, 1b-dihomo PGF_2α_ (*p* = 0.0117), PGF_2α_ 1,15-lactone (*p* = 0.0300), PGF_2α_ 1,11-lactone (*p* = 0.0474), and 5-trans PGF_2α_ (*p* = 0.0029), were downregulated in the MEL group compared to the CON group (Figures 4A–F). The abundance of the main metabolites of PGE_2_, including 13,14-dihydro-15-keto-PGE_2_ (*p* = 0.00002), 15-keto-PGE_2_ (*p* = 0.0494), 19(r)-hydroxy-PGE1 (*p* = 0.0006), and *ent*-prostaglandin E_2_ (*p* = 0.0034), significantly decreased after the intrauterine infusion of meloxicam (Figures 4G–J). Compared to the CON group, the abundance of PGI2 (*p* = 0.0008) and 6-keto-PGF1α (*p* = 0.00006), a stable metabolite of PGI2, were significantly downregulated in the MEL group (Figures 4K,L). Furthermore, 15-deoxy-Δ12,14-PGD2 (a main metabolite of PGD2) (*p* = 0.0044), 8-iso-prostaglandin A2 (*p* = 0.0032), and prostaglandin B2 (*p* = 0.0001) were also downregulated in the MEL group (Figures 4M–O). In addition to the major classes of the PGs, TXB2 was significantly downregulated in the MEL group (*p* = 0.0005) (Figure 4P). These results provide further evidence that intrauterine infusion of meloxicam inhibits prostaglandin synthesis.

### 3.4 The analysis of the differentially altered metabolites

**Figure 5:**
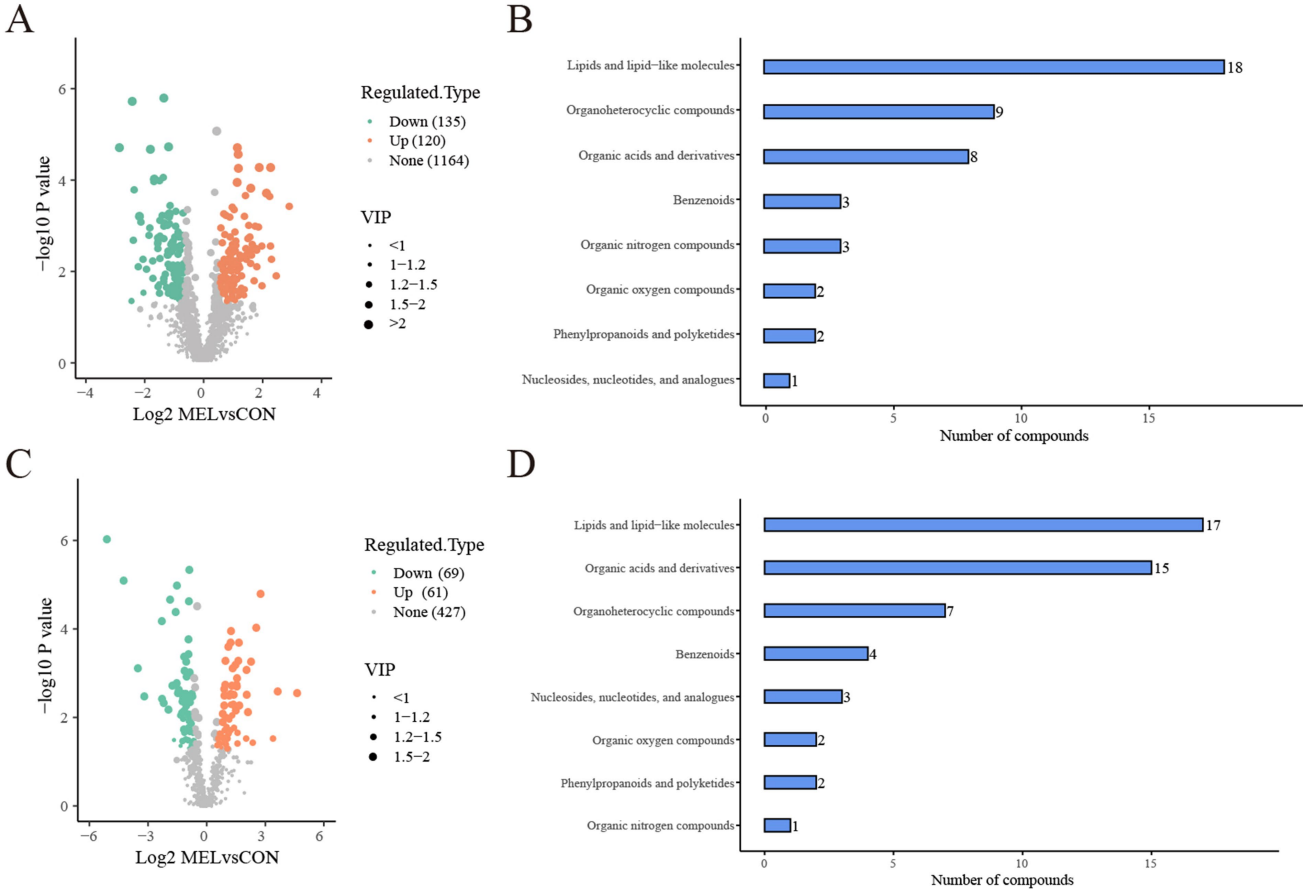
Analysis of the differentially altered metabolites between the CON and MEL groups. **(A)** Volcano plot of the differentially altered metabolites in the positive ion mode. **(B)** Bar graph showing the classification of the differential metabolites in the positive ion mode. **(C)** Volcano plot of the differentially altered metabolites group in the negative ion mode. **(D)** Bar graph showing the classification of the differential metabolites in the negative ion mode. CON, vehicle; MEL, meloxicam.

Based on the screening criteria, 255 differentially altered metabolites in the ULF were identified between the CON and MEL groups in the positive ion mode. Among these, 120 metabolites were upregulated, while 135 metabolites were downregulated (Figure 5A). In the negative ion mode, 130 differentially altered metabolites were identified, including 61 upregulated and 69 downregulated metabolites (Figure 5C). All differentially altered metabolites are presented in Supplementary Table S3. The differential metabolites were mainly categorized into lipids and lipid-like molecules, organic acids and derivatives, ogranoheterocyclic compounds, benzenoids, nucleosides, organic oxygen compounds, phenylpropanoids and polyketides, and organic nitrogen compounds (Figures 5B,D). In addition, the top 20 differentially altered metabolites in the positive and negative ion modes are shown in Table 1. These included benzalkonium, 2,8-dihydroxyquinoline-beta-d-glucuronide, Phe-Tyr, n-(1 h-indol-3-ylacetyl)glycine, phenylacetylglycine, tomatidine, alpha-n-phenylacetyl-l-glutamine, oxyquinoline, 5-methylcytidine, tiotropium, n1,n12-diacetylspermine, indacaterol, prostaglandin I2, 2-methylbutyroylcarnitine, n6,n6-dimethyladenosine, PGB2, acetylcarnitine, butyryl-l-carnitine, juvenile hormone I, and 3-hydroxybutyrylcarnitine in the positive mode, while hippuric acid, n-benzylformamide, inosine, salicyluric acid, daidzein, val-tyr, guanosine, p-acetamidophenyl, *β*-D-glucuronide, [[(4-hydroxyphenyl)acetyl]]aminoacetic acid, 4-pyridoxic acid, florfenicol, d-myoinositol 4-phosphate, 13,14-dihydro-15-ketoprostaglandin E2, 3-(cyclohexylamino)-2-hydroxy-1-propanesulfonic acid, TXB2, acamprosate, prostaglandin F1α., thyroxine, PGD1, 7z, 10z, 13z, 16z, and 19z-docosapentaenoic acid in the negative mode.

**Table 1:** List of the top 20 differentially altered metabolites in the uterine lumen fluid (ULF) between the CON and MEL groups in the positive and negative modes (VIP ≥ 1 and *p* < 0.05 and FC ≥ 1.5 or FC ≤ 0.667).

Metabolites	Fold change	*p*-value	VIP
Benzalkonium	7.761	0.00039	1.877
2,8-dihydroxyquinoline-beta-d-glucuronide	5.092	0.006	1.580
Phe-tyr	4.872	0.0002	1.976
n-(1 h-indol-3-ylacetyl) glycine	3.540	0.003	1.801
Phenylacetylglycine	3.392	0.017	1.531
Tomatidine	3.249	0.003	1.741
Alpha-n-phenylacetyl-l-glutamine	2.821	0.006	1.625
Oxyquinoline	2.788	0.003	1.827
5-methylcytidine	2.196	0.006	1.738
Tiotropium	2.113	0.021	1.378
n1, n12-diacetylspermine	0.197	0.002	1.946
Indacaterol	0.201	0.0002	1.952
Prostaglandin I2	0.236	0.001	1.850
2-methylbutyroylcarnitine	0.326	0.0001	2.033
n6, n6-dimethyladenosine	0.352	0.003	1.627
PGB2	0.366	0.0001	1.997
Acetylcarnitine	0.370	0.006	1.755
Butyryl-l-carnitine	0.389	0.001	1.962
Juvenile hormone i	0.394	0.016	1.492
3-hydroxybutyrylcarnitine	0.404	0.017	1.531
Hippuric acid	25.951	0.003	1.748
n-benzylformamide	12.950	0.002	1.595
Inosine	10.897	0.029	1.479
Salicyluric acid	7.087	0.00002	1.918
Daidzein	4.193	0.029	1.325
Val-tyr	3.195	0.0005	1.811
Guanosine	3.072	0.037	1.305
p-acetamidophenyl beta-D-glucuronide	3.061	0.001	1.702
[[(4-hydroxyphenyl)acetyl]amino]acetic acid	3.046	0.002	1.751
4-pyridoxic acid	2.730	0.003	1.646
Florfenicol	0.114	0.003	1.614
d-myoinositol 4-phosphate	0.215	0.004	1.742
13,14-dihydro-15-ketoprostaglandin E2	0.284	0.00002	1.933
3-(cyclohexylamino)-2-hydroxy-1-propanesulfonic acid	0.317	0.033	1.124
TXB2	0.364	0.00001	1.993
Acamprosate	0.401	0.043	1.092
Prostaglandin f1α	0.437	0.007	1.519
Thyroxine	0.448	0.011	1.444
PGD1	0.458	0.004	1.620
7z, 10z, 13z, 16z, 19z-docosapentaenoic acid	0.467	0.022	1.348

CON, vehicle; MEL, meloxicam; VIP, variable importance in projection; FC, fold change.

### 3.5 Metabolic pathway and network analysis of the differentially altered metabolites

**Figure 6:**
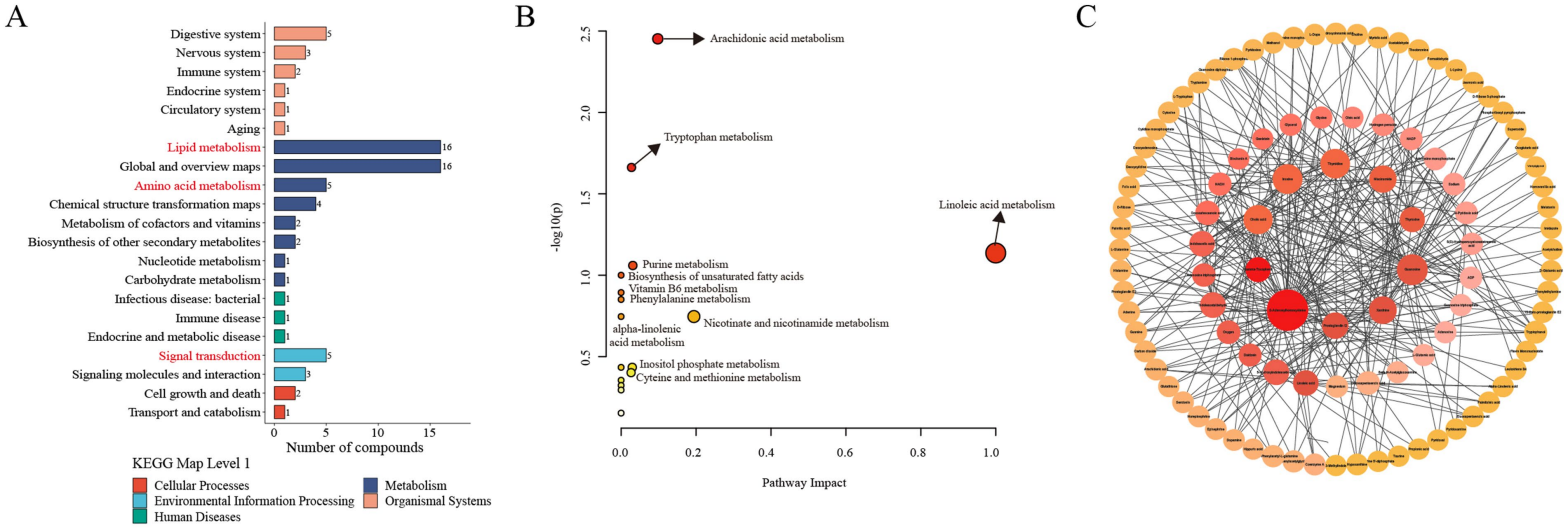
Metabolic pathway and network analysis of the differentially altered metabolites between the CON and MEL group. **(A)** KEGG enrichment analysis of the differential metabolites. **(B)** Metabolic pathway analysis using Metaboanalyst 6.0. The circles represent the different metabolic pathways; the arrow indicates significant changes in specific metabolites in the corresponding pathway; the size of the circle corresponds to the pathway impart score. **(C)** Metabolites network analysis using Metaboanalyst 6.0. and Cytoscape. The size of each dot represents the maximum clique centrality; the color of each dot represents the number of edges of the metabolite interacting with other metabolites. CON, vehicle; MEL, meloxicam; KEGG: Kyoto Encyclopedia of Genes and Genomes.

To investigate the functional changes induced by meloxicam, the differentially altered metabolites were subjected to KEGG metabolic pathway enrichment analysis. The results showed that the differentially altered metabolites mainly enriched in lipid metabolism, signal transduction, and amino acid metabolism (Figure 6A). To gain further insight into the alterations in the metabolic processes after the meloxicam treatment, the metabolic pathways of the differentially abundant metabolites were analyzed using MetaboAnalyst 6.0. The results showed that the differentially abundant metabolites were enriched in the following metabolism: arachidonic acid metabolism, tryptophan (Trp) metabolism, linoleic acid metabolism, purine metabolism, biosynthesis of unsaturated fatty acids, vitamin B6 metabolism, phenylalanine metabolism, alpha-linolenic acid metabolism, nicotinate and nicotinamide metabolism, inositol phosphate metabolism, and cysteine and methionine metabolism. Among these pathways, arachidonic acid metabolism, linoleic acid, and tryptophan metabolisms were identified as significant (Figure 6B).

To further identify hub metabolites in the differentially altered metabolites, we performed metabolite–metabolite network analyses using MetaboAnalyst 6.0. This analysis identified the top 10 core metabolites: S-adenosylhomocysteine (SAH), guanosine, inosine, thymidine, cholic acid, xanthine, niacinamide, prostaglandin I2, 5-hydroxyindoleacetic acid, and indoleacetaldehyde (Figure 6C).

### 3.6 Identification of the differential ULF metabolites associated with potential pregnancy

**Figure 7:**
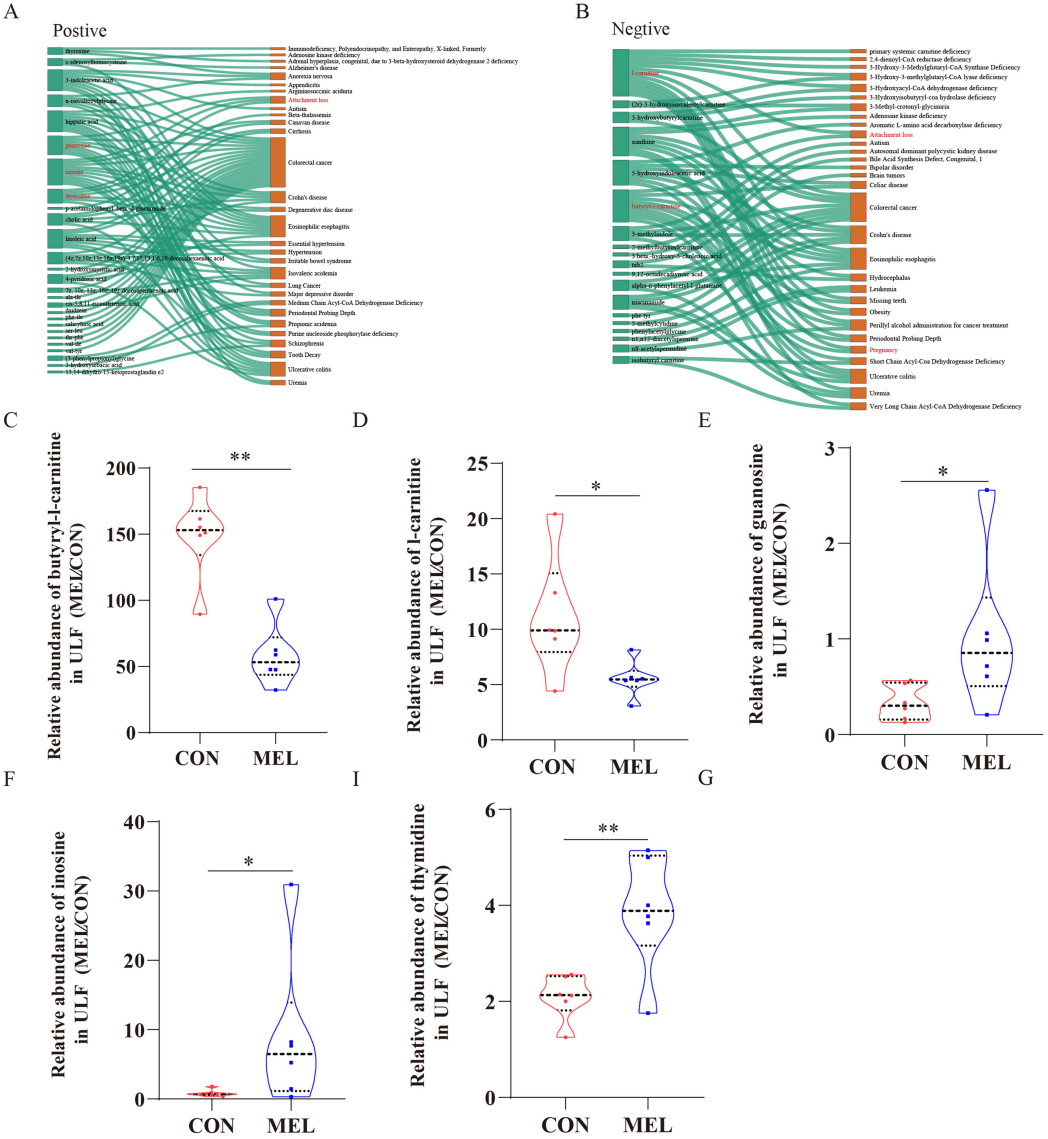
Screening of metabolites associated with pregnancy loss. Sankey diagrams demonstrating the correlation between the differential metabolites and the disease **(A)** in the positive ion mode, **(B)** in the negative ion mode. The relative abundance of **(C)** butyryl-l-carnitine, **(D)** l-carnitine, **(E)** guanosine, **(F)** inosine, and **(G)** thymidine in the uterine lumen fluid between the CON and MEL groups. CON, vehicle; MEL, meloxicam.

To investigate the potential biological significance of the metabolite changes, we utilized the KEGG database and the Human Metabolome Database (HMDB) to identify disease information associated with the differential metabolites. As shown in Figures 7A,B, five significantly differentially altered metabolites associated with pregnancy and attachment loss were identified and visualized using Sankey diagrams based on biological relevance and disease association analysis. These included guanosine (VIP = 1.305, FC = 3.072, *p* = 0.037; Figure 7C), inosine (VIP = 1.479, FC = 10.897, *p* = 0.029; Figure 7D), thymidine (VIP = 1.507, FC = 1.851, *p* = 0.012; Figure 7E), butyryl-l-carnitine (VIP = 1.962, FC = 0.389, *p* = 0.000631; Figure 7F), and l-carnitine (VIP = 1.342, FC = 0.494, *p* = 0.023; Figure 7G).

## 4 Discussion

During the preimplantation period, the endometrium secretes, transports, and/or synthesizes specific substances, regulated by coordinated maternal and conceptus signaling factors, which are essential for conceptus elongation and implantation (5, 10, 32, 33). From an *in vivo* perspective, this study provides a detailed biochemical analysis of the metabolic fingerprint of ULF induced by uterus-derived PGs on days 12–15 of the estrous cycle, which is considered to be the window for conceptus elongation initiation in cattle.

Meloxicam is a preferential inhibitor of COX-2 (34). Previous studies in sheep have shown that intrauterine perfusion of meloxicam reduces the levels of PGE_2_, PGF_2α,_ and 6-keto-PGF1α in uterine fluid (17, 35). In this study, we comprehensively analyzed the effects of meloxicam on PGs in the uterine fluid of dairy heifers. The abundances of PGI_2_ and its stable metabolite 6-keto-PGF1α and 15-deoxy-Δ12,14-PGD2 in the ULF were reduced after the intrauterine perfusion of meloxicam. 6-keto-PGF1α is the most abundant prostaglandin in cattle uterine fluid (19). Although the levels of PGE_2_ and PGF_2α_ were not significantly changed, the abundance of their major metabolites was reduced. Collectively, the intrauterine perfusion of meloxicam could reduce the content of the major PGs and metabolites in the ULF.

PGs mainly bind to receptors distributed in the conceptus and endometrium, which regulate endometrial responsiveness and subsequently affect conceptus development. After day 12 of pregnancy or the estrous cycle, the uterine lumen in cattle undergoes a metabolic shift, characterized by changes in amino acids, lipids, carbohydrates, cofactors and vitamins, nucleotides, and peptides (6, 11, 28). In this study, the intrauterine perfusion of meloxicam significantly altered the metabolic profile of the ULF, with differentially altered metabolites mainly enriched in lipid metabolism. During the onset of conceptus elongation, rapidly proliferating trophoblasts require lipids from ULF to synthesize membranes and serve as signaling molecules (29, 36). Lipid-related genes involved in conceptus elongation are associated with female fertility traits in dairy cows (37, 61).

In this study, several lipids and lipid-like molecules were identified, including docosanamide, 3*β*-hydroxy-5-cholenoic acid (3β-OH-5-Chln), auraptene, dinor-12-oxophytodienoic acid, l-arachidonoylcarnitine, isobutyryl carnitine, 5,8,11-eicosatriynoic acid, 5-hydroxyindoleacetic acid (5-HIAA), 9-OxoODE, 4β,5-epoxy-17β-hydroxy-5β-androstan-3-one, butyryl-l-carnitine, 2-methylbutyroylcarnitine, (2r)-3-hydroxyisovaleroylcarnitine, 3-hydroxybutyrylcarnitine, 2-hydroxymyristic acid, cholic acid, 11,14,17-eicosatrienoic acid, (z,z,z)-, linoleic acid, cis-5, 8, 11-eicosatrienoic acid, 9,12-octadecadiynoic acid, 17β-estradiol 3-β-d-glucuronide, 5 s-hydroperoxy-6e, 8z, 11z, 14z-eicosatetraenoic acid, 9 s-hydroxy-10e,12z,15z-octadecatrienoic acid, 12 s-hhtre, (4z, 7z, 10z, 13z, 16z, 19z)-4, 7, 10, 13, 16, 19-docosahexaenoic acid, and 7z, 10z, 13z, 16z, 19z-docosapentaenoic acid. Notably, cholic acid is a core metabolite that may serve as an indicator of fertility in dairy cows. It is the primary bile acid in domestic animals. Recent studies have found that cholic acid is present in the follicular fluid of both humans and bovines and is closely related to oocyte maturation and embryo development (38, 39). Cholic acid increases the expression of oxytocin receptors in the human endometrium (40). In ruminants, IFNT inhibits the upregulation of oxytocin receptors in the endometrial epithelia, further preventing the production of luteolytic PGF_2α_ pulses. Therefore, PGs may regulate endometrial responsiveness to embryo signals by influencing cholic acid in ULF.

In this study, we identified core metabolites, including S-adenosylhomocysteine (SAH), guanosine, inosine, thymidine, xanthine, niacinamide, and prostaglandin I2, which may serve as novel potential biomarkers for fertility in dairy cows. SAH is an important metabolic intermediate involved in methylation reactions and one-carbon metabolism, and it typically equilibrates with S-adenosylmethionine (SAM) *in vivo*. The SAM-to-SAH ratio serves as an indicator of methylation activity (41). Changes in endometrial and conceptus gene expression are likely partially attributed to alterations in DNA methylation in cattle (3, 42).

In this study, SAH was downregulated in the ULF. Downregulation of SAH is typically associated with an elevation in SAM, a key molecule in the methylation response, which directly affects embryonic development, placental function, and the normal progression of pregnancy. The abundance of SAM increases in response to elevated P_4_ levels in the ULF of dairy cows during the conceptus elongation period (28). Downregulation of SAH may help optimize the methylation status, which promotes normal embryo development and placental health, as well as the reduction of inflammation and oxidative stress (43). However, proper regulation of SAH levels is essential for a successful pregnancy and normal fetal development. The maintenance of normal one-carbon metabolism (OCM) ensures the synthesis of methyl donors, facilitating crucial processes such as DNA methylation, RNA methylation, and protein modification in cells that govern gene expression, cell proliferation, differentiation, and other physiological activities (44, 71). These results suggest that endometrium-derived PGs may affect maternal one-carbon metabolism in dairy heifers.

The pathway enrichment analysis revealed that tryptophan metabolism was significantly altered in the ULF. Dysregulation of maternal amino acid Trp metabolism is associated with adverse pregnancy outcomes (45). Trp is essential for maternal protein synthesis, embryo growth, and development (46). Trp metabolism is also involved in one-carbon metabolism (OCM) by providing substrates, cofactors, and methyl groups. Moreover, OCM is crucial for early embryo development and plays an important role in epigenetic modifications, as well as the biosynthesis of DNA, proteins, and lipids (47, 48). 5-HIAA and indoleacetaldehyde are involved in TRP metabolism and are also core components of differentially altered metabolites. Rapidly proliferating trophectoderm cells in sheep rely on OCM for the production of formate required for nucleic acid synthesis during the peri-implantation period of pregnancy (49). This further suggests that PGs may affect maternal one-carbon metabolism.

Tryptophan metabolism is closely associated with immune tolerance during pregnancy. PGs play an important role in maternal immune tolerance during pregnancy. The prevention of fetal rejection involves the suppression of T-cell activity through the depletion of TRP after the induction of cytokines, which leads to the activation of indoleamine 2,3-dioxygenase (IDO), an extrahepatic enzyme responsible for TRP degradation (50). In mouse macrophages, the tryptophan metabolite I3A hinders the expression of inflammatory cytokines and inhibits the migration of cells toward chemokines (51, 52). In this study, the tryptophan metabolite I3A was significantly downregulated, which may have affected the migration of embryonic trophoblasts. These results further suggest that Trp metabolism may play an important role in the effects of PGs on maternal immune tolerance.

In this study, the abundance of linoleic acid (LA) and *α*-linolenic acid (ALA) in the ULF was significantly downregulated after the meloxicam treatment, while the linoleic acid metabolism pathway showed significant enrichment. Supplementing beef cattle with linoleic acid-rich soybean oil post-artificial insemination increased pregnancy rates by 30% and elevated plasma LA and P_4_ concentrations (53, 54). In addition, rumen-protected conjugated linoleic acid positively affects beef cow reproduction (55, 56). It is worth noting that ALA and LA act as optimal ligands for PPARs and can activate them (57). PPARs, particularly PPARγ, significantly contribute to the regulation of embryonic development, which coordinates lipid-related gene expression in trophectoderm cells (58, 63–67). Therefore, endometrium-derived PGs may affect the content of long-chain polyunsaturated fatty acids in ULF, which regulate conceptus elongation by binding to PPARs in trophoblast cells.

LA, a competitive inhibitor of arachidonic acid metabolism and a precursor for PG synthesis, is increased in the endometrium of pregnant cattle (59). Intrauterine administration of LA or ALA in cows between days 12 and 21 promotes PGE_2_ production while inhibiting PGF_2α_ synthesis in the endometrium, which is beneficial for pregnancy maintenance (59). Linoleic acid is a substrate for enzymes involved in extracellular matrix (ECM) remodeling (60). A previous study found that pregnancy loss in subfertile heifers was associated with ECM remodeling, and excessive ECM can inhibit embryonic adhesion to the endometrium (3). Consumption of conjugated linoleic acid (CLA) in healthy and cancerous rats has been found to reduce the serum levels of MMP9. Collectively, these findings indicate that LA in ULF may regulate dynamic endometrial remodeling during early pregnancy in an autocrine manner.

Although this estrous cycle model is valuable for understanding the multifactorial phenomenon of conceptus elongation and identifying candidate metabolites involved in regulating conceptus elongation and implantation in ruminants, drawing biologically meaningful conclusions about conceptus elongation remains a significant challenge in the estrous cycle. Therefore, future studies will need to integrate approaches such as lentiviral vectors and antisense oligodeoxynucleotides in pregnant cows to elucidate the mechanistic roles of specific factors governing conceptus elongation and uterine receptivity.

## 5 Conclusion

This study revealed alterations in the metabolites of the uterine fluid induced by uterus-derived PGs during diestrus in dairy heifers. These changes primarily involved lipids, amino acids, and nucleotides, with significant enrichment in tryptophan metabolism, linoleic acid metabolism, and purine metabolism, all of which are associated with one-carbon metabolism. As signaling molecules during early pregnancy in ruminants, PGs may regulate physiological metabolic shifts in ULF after day 12 by influencing one-carbon metabolism. These findings deepen our understanding of conceptus elongation *in vivo* and offer new strategies for enhancing fertility in domestic animals.

## Data Availability

The original contributions presented in the study are included in the article/Supplementary material, further inquiries can be directed to the corresponding authors.

## Footnotes

^1^ https://metlin.scripps.edu

^2^ http://www.metaboanalyst.ca

## References

[1] Cabrera VE. Economics of fertility in high-yielding dairy cows on confined TMR systems. Animal. (2014) 8:211–21. doi: 10.1017/S1751731114000512, PMID: 24679357

[2] Berg DK van Leeuwen J Beaumont S Berg M Pfeffer PL. Embryo loss in cattle between days 7 and 16 of pregnancy. Theriogenology. (2010) 73:250–60. doi: 10.1016/j.theriogenology.2009.09.005, PMID: 19880168

[3] Moraes JGN Behura SK Geary TW Hansen PJ Neibergs HL Spencer TE. Uterine influences on conceptus development in fertility-classified animals. Proc Natl Acad Sci USA. (2018) 115:E1749–e1758. doi: 10.1073/pnas.1721191115, PMID: 29432175 PMC5828633

[4] Spencer TE Forde N Lonergan P. The role of progesterone and conceptus-derived factors in uterine biology during early pregnancy in ruminants. J Dairy Sci. (2016) 99:5941–50. doi: 10.3168/jds.2015-10070, PMID: 26387021

[5] Brooks K Burns G Spencer TE. Conceptus elongation in ruminants: roles of progesterone, prostaglandin, interferon tau and cortisol. J Anim Sci Biotechnol. (2014) 5:53. doi: 10.1186/2049-1891-5-53, PMID: 25810904 PMC4373033

[6] Simintiras CA Sánchez JM McDonald M Lonergan P. Progesterone alters the bovine uterine fluid lipidome during the period of elongation. Reproduction. (2019) 157:399–411. doi: 10.1530/REP-18-0615 30763281

[7] Gray CA Taylor KM Ramsey WS Hill JR Bazer FW Bartol FF. Endometrial glands are required for preimplantation conceptus elongation and survival. Biol Reprod. (2001) 64:1608–13. doi: 10.1095/biolreprod64.6.1608, PMID: 11369585

[8] Betteridge KJ Eaglesome MD Randall GC Mitchell D. Collection, description and transfer of embryos from cattle 10-16 days after oestrus. J Reprod Fertil. (1980) 59:205–16. doi: 10.1530/jrf.0.0590205, PMID: 7401037

[9] Gray CA Burghardt RC Johnson GA Bazer FW Spencer TE. Evidence that absence of endometrial gland secretions in uterine gland knockout ewes compromises conceptus survival and elongation. Reproduction. (2002) 124:289–300. doi: 10.1530/rep.0.1240289 12141942

[10] Bazer FW Wu G Johnson GA Kim J Song G. Uterine histotroph and conceptus development: select nutrients and secreted phosphoprotein 1 affect mechanistic target of rapamycin cell signaling in ewes. Biol Reprod. (2011) 85:1094–107. doi: 10.1095/biolreprod.111.094722, PMID: 21865556

[11] Simintiras CA Drum JN Liu H Sofia Ortega M Spencer TE. Uterine lumen fluid is metabolically semi-autonomous. Commun Biol. (2022) 5:191. doi: 10.1038/s42003-022-03134-0, PMID: 35233029 PMC8888695

[12] Kelleher AM DeMayo FJ Spencer TE. Uterine glands: developmental biology and functional roles in pregnancy. Endocr Rev. (2019) 40:1424–45. doi: 10.1210/er.2018-00281, PMID: 31074826 PMC6749889

[13] Moraes JGN Behura SK Bishop JV Hansen TR Geary TW Spencer TE. Analysis of the uterine lumen in fertility-classified heifers: II. Proteins and metabolites†. Biol Reprod. (2020) 102:571–87. doi: 10.1093/biolre/ioz197, PMID: 31616912 PMC7331878

[14] Spencer TE Hansen TR. Implantation and establishment of pregnancy in ruminants. Adv Anat Embryol Cell Biol. (2015) 216:105–35. doi: 10.1007/978-3-319-15856-3_7 26450497

[15] Forde N Carter F Spencer TE Bazer FW Sandra O Mansouri-Attia N. Conceptus-induced changes in the endometrial transcriptome: how soon does the cow know she is pregnant? Biol Reprod. (2011) 85:144–56. doi: 10.1095/biolreprod.110.090019, PMID: 21349821

[16] Arosh JA Banu SK McCracken JA. Novel concepts on the role of prostaglandins on luteal maintenance and maternal recognition and establishment of pregnancy in ruminants. J Dairy Sci. (2016) 99:5926–40. doi: 10.3168/jds.2015-10335, PMID: 27179861

[17] Dorniak P Bazer FW Spencer TE. Prostaglandins regulate conceptus elongation and mediate effects of interferon tau on the ovine uterine endometrium. Biol Reprod. (2011) 84:1119–27. doi: 10.1095/biolreprod.110.089979, PMID: 21270428

[18] Spencer TE Forde N Dorniak P Hansen TR Romero JJ Lonergan P. Conceptus-derived prostaglandins regulate gene expression in the endometrium prior to pregnancy recognition in ruminants. Reproduction. (2013) 146:377–87. doi: 10.1530/REP-13-0165, PMID: 23966582 PMC3791335

[19] Ulbrich SE Schulke K Groebner AE Reichenbach HD Angioni C Geisslinger G. Quantitative characterization of prostaglandins in the uterus of early pregnant cattle. Reproduction. (2009) 138:371–82. doi: 10.1530/REP-09-0081, PMID: 19470711

[20] Moraes JGN Behura SK Geary TW Spencer TE. Analysis of the uterine lumen in fertility-classified heifers: I. Glucose, prostaglandins, and lipids†. Biol Reprod. (2020) 102:456–74. doi: 10.1093/biolre/ioz191, PMID: 31616913 PMC7331873

[21] Juengel JL Mosaad EMO Mitchell MD Phyn CVC French MC Meenken ED. Relationships between prostaglandin concentrations, a single nucleotide polymorphism in HSD17B12, and reproductive performance in dairy cows. J Dairy Sci. (2022) 105:4643–52. doi: 10.3168/jds.2021-21298, PMID: 35221066

[22] Jaureguiberry M Madoz LV Quintana S Marín M Burucúa M Tizzano M. Endometrial expression of key genes related to fertility in repeat breeder and non-repeat breeder cows. Reprod Domest Anim. (2020) 55:1660–4. doi: 10.1111/rda.13841, PMID: 33047395

[23] Smith WL. Nutritionally essential fatty acids and biologically indispensable cyclooxygenases. Trends Biochem Sci. (2008) 33:27–37. doi: 10.1016/j.tibs.2007.09.013, PMID: 18155912

[24] Arosh JA Banu SK Kimmins S Chapdelaine P Maclaren LA Fortier MA. Effect of interferon-tau on prostaglandin biosynthesis, transport, and signaling at the time of maternal recognition of pregnancy in cattle: evidence of polycrine actions of prostaglandin E2. Endocrinology. (2004) 145:5280–93. doi: 10.1210/en.2004-0587, PMID: 15308607

[25] Simmons RM Satterfield MC Welsh TH Jr Bazer FW Spencer TE. HSD11B1, HSD11B2, PTGS2, and NR3C1 expression in the peri-implantation ovine uterus: effects of pregnancy, progesterone, and interferon tau. Biol Reprod. (2010) 82:35–43. doi: 10.1095/biolreprod.109.079608, PMID: 19696010

[26] Erdem H Guzeloglu A. Effect of meloxicam treatment during early pregnancy in Holstein heifers. Reprod Domest Anim. (2010) 45:625–8. doi: 10.1111/j.1439-0531.2008.01317.x, PMID: 19144041

[27] O'Neil EV Brooks K Burns GW Ortega MS Denicol AC Aguiar LH. Prostaglandin-endoperoxide synthase 2 is not required for preimplantation ovine conceptus development in sheep. Mol Reprod Dev. (2020) 87:142–51. doi: 10.1002/mrd.23300, PMID: 31746519

[28] Simintiras CA Sánchez JM McDonald M Lonergan P. The biochemistry surrounding bovine conceptus elongation†. Biol Reprod. (2019) 101:328–37. doi: 10.1093/biolre/ioz101, PMID: 31181571

[29] King K Ticiani E Sprícigo JFW Carvalho MR Mion B Bertolini M. Dynamics of lipid droplets in the endometrium and fatty acids and oxylipins in the uterine lumen, blood, and milk of lactating cows during diestrus. J Dairy Sci. (2021) 104:3676–92. doi: 10.3168/jds.2020-19196, PMID: 33455794

[30] Kokla M Virtanen J Kolehmainen M Paananen J Hanhineva K. Random forest-based imputation outperforms other methods for imputing LC-MS metabolomics data: a comparative study. BMC Bioinformatics. (2019) 20:492. doi: 10.1186/s12859-019-3110-0, PMID: 31601178 PMC6788053

[31] Pang Z Chong J Zhou G de Lima Morais DA Chang L Barrette M. MetaboAnalyst 5.0: narrowing the gap between raw spectra and functional insights. Nucleic Acids Res. (2021) 49:W388–w396. doi: 10.1093/nar/gkab382, PMID: 34019663 PMC8265181

[32] Forde N McGettigan PA Mehta JP O'Hara L Mamo S Bazer FW. Proteomic analysis of uterine fluid during the pre-implantation period of pregnancy in cattle. Reproduction. (2014) 147:575–87. doi: 10.1530/REP-13-0010, PMID: 24478148

[33] Martins T Sponchiado M Silva F Estrada-Cortés E Hansen PJ Peñagaricano F. Progesterone-dependent and progesterone-independent modulation of luminal epithelial transcription to support pregnancy in cattle. Physiol Genomics. (2022) 54:71–85. doi: 10.1152/physiolgenomics.00108.2021, PMID: 34890509 PMC8791843

[34] Kovács L Kézér FL Ruff F Samardzija M Szenci O. Single-dose meloxicam treatment improves standing ability of low-vitality dairy calves. J Dairy Sci. (2022) 105:1618–24. doi: 10.3168/jds.2021-20704, PMID: 34799121

[35] Dorniak P Bazer FW Wu G Spencer TE. Conceptus-derived prostaglandins regulate endometrial function in sheep. Biol Reprod. (2012) 87:1–7. doi: 10.1095/biolreprod.112.100487, PMID: 22517622

[36] Ribeiro ES Santos JE Thatcher WW. Role of lipids on elongation of the preimplantation conceptus in ruminants. Reproduction. (2016) 152:R115–26. doi: 10.1530/REP-16-0104, PMID: 27335133

[37] Abdollahi-Arpanahi R Carvalho MR Ribeiro ES Peñagaricano F. Association of lipid-related genes implicated in conceptus elongation with female fertility traits in dairy cattle. J Dairy Sci. (2019) 102:10020–9. doi: 10.3168/jds.2019-17068, PMID: 31477299

[38] Blaschka C Sánchez-Guijo A Wudy SA Wrenzycki C. Profile of bile acid subspecies is similar in blood and follicular fluid of cattle. Vet Med Sci. (2020) 6:167–76. doi: 10.1002/vms3.217, PMID: 31713347 PMC7196682

[39] Nagy RA van Montfoort APA Dikkers A van Echten-Arends J Homminga I Land JA. Presence of bile acids in human follicular fluid and their relation with embryo development in modified natural cycle IVF. Hum Reprod. (2015) 30:1102–9. doi: 10.1093/humrep/dev034 25753582

[40] Germain AM Kato S Carvajal JA Valenzuela GJ Valdes GL Glasinovic JC. Bile acids increase response and expression of human myometrial oxytocin receptor. Am J Obstet Gynecol. (2003) 189:577–82. doi: 10.1067/S0002-9378(03)00545-3, PMID: 14520238

[41] Crouse MS Freetly HC Lindholm-Perry AK Neville BW Oliver WT Lee RT. One-carbon metabolite supplementation to heifers for the first 14 d of the estrous cycle alters the plasma and hepatic one-carbon metabolite pool and methionine-folate cycle enzyme transcript abundance in a dose-dependent manner. J Anim Sci. (2023) 101:skac419. doi: 10.1093/jas/skac419, PMID: 36566452 PMC9890446

[42] Walker CG Littlejohn MD Meier S Roche JR Mitchell MD. DNA methylation is correlated with gene expression during early pregnancy in *Bos taurus*. Physiol Genomics. (2013) 45:276–86. doi: 10.1152/physiolgenomics.00145.2012, PMID: 23386203

[43] Shojaei Saadi HA Gagné D Fournier É Baldoceda Baldeon LM Sirard MA Robert C. Responses of bovine early embryos to S-adenosyl methionine supplementation in culture. Epigenomics. (2016) 8:1039–60. doi: 10.2217/epi-2016-0022, PMID: 27419740

[44] McFadden JW Girard CL Tao S Zhou Z Bernard JK Duplessis M. Symposium review: one-carbon metabolism and methyl donor nutrition in the dairy cow. J Dairy Sci. (2020) 103:5668–83. doi: 10.3168/jds.2019-17319, PMID: 32278559

[45] van Zundert SKM van Egmond NCM van Rossem L Willemsen SP Griffioen PH van Schaik RHN. First trimester maternal tryptophan metabolism and embryonic and fetal growth: the Rotterdam Periconceptional cohort (predict study). Hum Reprod. (2024) 39:912–22. doi: 10.1093/humrep/deae046, PMID: 38498837 PMC11063566

[46] Badawy AA. Tryptophan metabolism, disposition and utilization in pregnancy. Biosci Rep. (2015) 35:e00261. doi: 10.1042/BSR20150197, PMID: 26381576 PMC4626867

[47] Kalhan SC. One carbon metabolism in pregnancy: impact on maternal, fetal and neonatal health. Mol Cell Endocrinol. (2016) 435:48–60. doi: 10.1016/j.mce.2016.06.006, PMID: 27267668 PMC5014566

[48] Pentieva K Caffrey A Duffy B Ward M Clements M Kerr M. B-vitamins and one-carbon metabolism during pregnancy: health impacts and challenges. Proc Nutr Soc. (2024):1–15. doi: 10.1017/S0029665124004865 39311046

[49] Halloran KM Stenhouse C Moses RM Kramer AC Sah N Seo H. The ovine conceptus utilizes extracellular serine, glucose, and fructose to generate formate via the one carbon metabolism pathway. Amino Acids. (2023) 55:125–37. doi: 10.1007/s00726-022-03212-x, PMID: 36383272

[50] Munn DH Zhou M Attwood JT Bondarev I Conway SJ Marshall B. Prevention of allogeneic fetal rejection by tryptophan catabolism. Science. (1998) 281:1191–3. doi: 10.1126/science.281.5380.1191, PMID: 9712583

[51] Krishnan S Ding Y Saedi N Choi M Sridharan GV Sherr DH. Gut microbiota-derived tryptophan metabolites modulate inflammatory response in hepatocytes and macrophages. Cell Rep. (2018) 23:1099–111. doi: 10.1016/j.celrep.2018.03.109, PMID: 29694888 PMC6392449

[52] Ding Y Yanagi K Yang F Callaway E Cheng C Hensel ME. Oral supplementation of gut microbial metabolite indole-3-acetate alleviates diet-induced steatosis and inflammation in mice. eLife. (2024) 12:12. doi: 10.7554/eLife.87458.3, PMID: 38412016 PMC10942630

[53] Lopes CN Cooke RF Reis MM Peres RF Vasconcelos JL. Strategic supplementation of calcium salts of polyunsaturated fatty acids to enhance reproductive performance of *Bos indicus* beef cows. J Anim Sci. (2011) 89:3116–24. doi: 10.2527/jas.2011-3909, PMID: 21622871

[54] Lopes CN Scarpa AB Cappellozza BI Cooke RF Vasconcelos JL. Effects of rumen-protected polyunsaturated fatty acid supplementation on reproductive performance of *Bos indicus* beef cows. J Anim Sci. (2009) 87:3935–43. doi: 10.2527/jas.2009-2201, PMID: 19684273

[55] Rodney RM Celi P Scott W Breinhild K Lean IJ. Effects of dietary fat on fertility of dairy cattle: a meta-analysis and meta-regression. J Dairy Sci. (2015) 98:5601–20. doi: 10.3168/jds.2015-9528, PMID: 26094218

[56] Cooke RF. Early career achievement award: supplementing omega-6 fatty acids to enhance early embryonic development and pregnancy establishment in Bos indicus and *B. taurus* beef cows. J Anim Sci. (2019) 97:485–95. doi: 10.1093/jas/sky414, PMID: 30351357 PMC6313120

[57] Echeverría F Ortiz M Valenzuela R Videla LA. Long-chain polyunsaturated fatty acids regulation of PPARs, signaling: relationship to tissue development and aging. Prostaglandins Leukot Essent Fatty Acids. (2016) 114:28–34. doi: 10.1016/j.plefa.2016.10.001, PMID: 27926461

[58] Peixoto PM Bromfield JJ Ribeiro ES Santos JEP Thatcher WW Bisinotto RS. Transcriptome changes associated with elongation of bovine conceptuses I: differentially expressed transcripts in the conceptus on day 17 after insemination. J Dairy Sci. (2023) 106:9745–62. doi: 10.3168/jds.2023-23398, PMID: 37641295

[59] Sakumoto R Hayashi KG Iga K. Direct effects of linoleic and linolenic acids on bovine uterine function using in vivo and in vitro studies. J Reprod Dev. (2022) 68:62–7. doi: 10.1262/jrd.2021-107, PMID: 34803128 PMC8872745

[60] Bueno Cordeiro Maldonado M de Castro LV de Oliveira BL Feltrin IR Mendes AF Rocha CC. Conjugated linoleic acid supplementation changes prostaglandin concentration ratio and alters the expression of genes involved in maternal-fetal recognition from bovine trophoblast cells in vitro. Theriogenology. (2023) 206:87–95. doi: 10.1016/j.theriogenology.2023.04.003, PMID: 37201299

[61] Ribeiro ES Greco LF Bisinotto RS Lima FS Thatcher WW Santos JE. Biology of preimplantation conceptus at the onset of elongation in dairy cows. Biol Reprod. (2016) 94:97. doi: 10.1095/biolreprod.115.134908, PMID: 26935601

[62] Geary TW Burns GW Moraes JG Moss JI Denicol AC Dobbs KB. Identification of beef heifers with superior uterine capacity for pregnancy. Biol Reprod. (2016) 95:47. doi: 10.1095/biolreprod.116.141390, PMID: 27417907 PMC5029478

[63] Forman BM Tontonoz P Chen J Brun RP Spiegelman BM Evans RM. 15-deoxy-delta 12, 14-prostaglandin J2 is a ligand for the adipocyte determination factor PPAR gamma. Cell. (1995) 83:803–12. doi: 10.1016/0092-8674(95)90193-0 8521497

[64] Lim H Gupta RA Ma WG Paria BC Moller DE Morrow JD. Cyclo-oxygenase-2-derived prostacyclin mediates embryo implantation in the mouse via PPARdelta. Genes Dev. (1999) 13:1561–74. doi: 10.1101/gad.13.12.1561, PMID: 10385625 PMC316805

[65] Pérez-Gómez A González-Brusi L Flores-Borobia I De Los M Reyes N Toledano-Díaz A. PPARG is dispensable for bovine embryo development up to tubular stages†. Biol Reprod. (2024) 111:557–66. doi: 10.1093/biolre/ioae083, PMID: 38832705 PMC11402522

[66] Brooks KE Burns GW Spencer TE. Peroxisome proliferator activator receptor gamma (PPARG) regulates conceptus elongation in sheep. Biol Reprod. (2015) 92:42. doi: 10.1095/biolreprod.114.123877, PMID: 25519185

[67] McGraw MS Rajput SK Daigneault BW. PPAR-gamma influences developmental competence and trophectoderm lineage specification in bovine embryos. Reproduction. (2024):167:e230334. doi: 10.1530/REP-23-0334 38301360

[68] Nakamura MT Yudell BE Loor JJ. Regulation of energy metabolism by long-chain fatty acids. Prog Lipid Res. (2014) 53:124–44. doi: 10.1016/j.plipres.2013.12.001 24362249

[69] Zhen Y Krausz KW Chen C Idle JR Gonzalez FJ. Metabolomic and genetic analysis of biomarkers for peroxisome proliferator-activated receptor alpha expression and activation. Mol Endocrinol. (2007) 21:2136–51. doi: 10.1210/me.2007-0150, PMID: 17550978 PMC2084472

[70] Forde N Carter F Fair T Crowe MA Evans AC Spencer TE. Progesterone-regulated changes in endometrial gene expression contribute to advanced conceptus development in cattle. Biol Reprod. (2009) 81:784–94. doi: 10.1095/biolreprod.108.074336, PMID: 19553605

[71] Rong S Fu Y Zhao Y Zhu W Mu L. How purine metabolites impact reproduction. Trends Endocrinol Metab. (2024). doi: 10.1016/j.tem.2024.08.010, PMID: 39271435

