# Supplementary material for: Metabolomic effects of intrauterine meloxicam perfusion on histotroph in dairy heifers during diestrus
Source: Front Vet Sci. 2025 Mar 18;12:1528530. doi: 10.3389/fvets.2025.1528530 (PMC11959509; doi:10.3389/fvets.2025.1528530)
Supplement: Supplementary file 1 [file Data_Sheet_1.ZIP › supplemential table/Table S1.docx]

| Table 1: Ultrasonographic assessment of ovarian structures in heifers on different timepoints during the estrous cycles. | | | | | |
| --- | --- | --- | --- | --- | --- |
| group | heifers | | US at day 0 | US at day 7 | US at day 15 |
| CON | 1 | | R: 2.3 cm follicle  L: several<0.5 cm follicles | R: 2.7 cm cavitary CL (CL_cav_)  L: several<0.5 cm follicles | R: 2.6 cm CL  L: 1.3 cm follicle |
|  | 2 | R: several<0.5 cm follicles  L: 2.1 cm follicles | | R: several<0.5 cm follicles  L: 2.4 cm CL_cav_ | R: 1.0 cm follicle  L: 2.7 cm CL |
|  | 3 | R: 1.9 cm follicle  L: several<0.5 cm follicles | | R: 2.8 cm CL_cav_  L: several<0.5 cm follicles | R: 3.0 cm CL  L: 2.3 cm follicle |
|  | 4 | R: 2.0 cm follicle  L: several<0.5 cm follicles | | R: 3.0 cm CL_cav_  L: several<0.5 cm follicles | R: 3.6 cm CL_cav_  L: 2.6 cm follicle |
|  | 5 | R: several<0.5 cm follicles  L: 2.1 cm follicle | | R: several<0.5 cm follicles  L: 2.7 cm CL_cav_ | R: 1.8 cm follicle  L: 3.3 cm CL |
|  | 6 | R: several<0.5 cm follicles  L: 2.2 cm follicle | | R: several<0.5 cm follicles  L: 2.9 cm CL_cav_ | R: 2.5 cm follicle  L: 3.0 cm CL |
| MEL | 1 | R: 1.5 cm follicle  L: several<0.5 cm follicles | | R: 2.7 cm CL_cav_  L: several<0.5 cm follicles | R: 3.3 cm CL  L: 1.9 cm follicle |
|  | 2 | R: several<0.5 cm follicles  L: 2.0 cm follicle | | R: several<0.5 cm follicles  L: 2.5 cm CL_cav_ | R: 2.5 cm follicle  L: 3.0 cm CL |
|  | 3 | R: 2.2 cm follicle  L: several<0.5 cm follicles | | R: 3.1 cm CL_cav_  L: several<0.5 cm follicles | R: 3.7 cm CL_cav_  L: 2.4 cm follicle |
|  | 4 | R: several<0.5 cm follicles  L:1.8 cm follicle | | R: several<0.5 cm follicles  L: 2.5 cm CL_cav_ | R: 2.1 cm follicle  L: 2.9 cm CL |
|  | 5 | R: several<0.5 cm follicles  L: 2.2 cm follicle. | | R: several<0.5 cm follicles  L: 2.6 cm CL_cav_ | R: 1.5 cm follicle  L: 3.3 cm CL |
|  | 6 | R: 2.4 cm follicle  L: several<0.5 cm follicles | | R: 2.6 cm CL_cav_  L: several<0.5 cm follicles | R: 2.9 cm CL  L: 1.6 cm follicle |

US: ultrasonography; CL: corpus luteum; R: right; L: left; CL_cav_: cavitary corpus luteum.
